# Association between quality antenatal care and low birth weight in Rwanda: a cross-sectional study design using the Rwanda demographic and health surveys data

**DOI:** 10.1186/s12913-023-09482-9

**Published:** 2023-05-30

**Authors:** Gérard Uwimana, Mohamed Elhoumed, Mitslal Abrha Gebremedhin, Mougni Mohamed Azalati, Lin Nan, Lingxia Zeng

**Affiliations:** 1 Department of Epidemiology and Biostatistics, School of Public Health, Xi’an Jiaotong University Health Science Center, No 76 West Yanta Road, Xi’an, 710061 Shaanxi Province People’s Republic of China; 2 National Institute of Public Health Research (INRSP), BP. 695, Nouakchott, Mauritania; 3 Key Laboratory of Environment and Genes Related to Diseases, Xi’an Jiaotong University, Ministry of Education, Xi’an, 710061 Shaanxi P.R. China

**Keywords:** Quality of antenatal care, Low birth weight, Rwanda, DHS

## Abstract

**Background:**

Low birth weight (LBW) is an important factor influencing infant morbidity and mortality. Pregnant women should receive a variety of interventions during antenatal care (ANC) that are crucial in improving birth weight. ANC visits alone do not promise that women have received all recommended antenatal services. However, there are limited evidence of the relationship between ANC quality and LBW in Rwanda. Therefore, the purpose of this study was to assess the association between quality ANC and LBW along with the factors influencing LBW and how quality ANC affects LBW in Rwandan pregnant women.

**Methods:**

The Demographic and Health Surveys (DHS) are cross-sectional, nationally representative household surveys that collect population, health, and nutrition. In this Study we used three waves of Rwanda Demographic and Health Surveys 2010,2014-5 and 2019-20. A total of 16,144 women aged 15 to 49 years who had live births in the five years preceding each survey were included in this study. A stratified two-stage sampling methods was used to select the participants. The first stage involves selecting clusters (villages) from a list of all clusters in the country. The second stage involves selecting households within each cluster. A survey adjusted for clusters at multiple level and a bivariate and multivariable logistic regression was used to estimate adjusted odds ratios(aOR) and 95% confidence intervals to assess the association between the outcome and independent variables.

**Results:**

The utilization of a high-quality ANC increased slightly over the three survey years and LBW had a slow decline. Out of 5813 women;201(3.45%) had high-quality ANC in the 2010 survey, and out of 5813 newborns,180(3.10%) were LBW. Out of 5404 women;492(9.11%) had high-quality ANC in 2015, and out of 5404 newborns,151(2.79% were LBW). Out of 5203 women,776(14.92%) had high-quality ANC in the 2020 survey year, and out of the 5206 newborns,139(2.67%) were LBW. In multivariable analysis, at a borderline limit high quality ANC was negatively associated with LBW(aOR:0.67;95%CI:0.43,1.05) compared to low-quality ANC. Higher birth orders of the newborn were negatively associated with LBW (aOR:0.63;95%CI:0.49,0.82 and aOR:0.44;95%CI:0.32,0.61 for 2nd -3rd and 4th and above respectively) compared to 1st orders newborn. Newborns from rich households were less likely to experience LBW than those from poor households (aOR:0.71;95%CI:0.55,0.91). Female newborns were associated with an increase of LBW (aOR:1.43;95% CI:1.18,1.73) than male newborns.

**Conclusion:**

The findings confirm the fundamental importance of a high-quality ANC on LBW. The findings could be utilized to develop monitoring strategies and assess pregnancy health assistance programs with a focus on LBW reduction.

**Supplementary Information:**

The online version contains supplementary material available at 10.1186/s12913-023-09482-9.

## Background

According to the World Health Organization (WHO), low birth weight (LBW) is defined as an infant’s birth weight of less than 2500 g, regardless of gestational age or other factors [1]. LBW causes 60–80% of all newborn deaths worldwide and increases the risk of mortality by 20–30 times [2, 3]. Immediately following delivery and for the first year of life, surviving newborns are more susceptible to pathological diseases like infection [4]. Later-life morbidity is also linked to LBW, including psychosocial disorders [5], poor cognitive function [6], coronary heart disease [7], and non-insulin-dependent diabetes [8].

Low and middle-income countries(LMICs) and especially Sub-Saharan Africa(SSA) countries are affected by a higher rate of LBW, WHO estimates that roughly 95.6% of the more than 20 million LBW babies (representing 15.5% of all live births) are in LMICs [9], estimated LBW levels in SSA are at 15% [10, 11]. This is related to the inadequate health infrastructure and permeable social support systems found in the majority of developing nations, which have a detrimental effect on health outcomes. Several studies on LBW in SSA have demonstrated that adherence to antenatal care (ANC) services, maternal body mass index(BMI), the receipt of iron and folic acid during pregnancy, gender of the newborn, demographic and socioeconomic factors such as household wealth index, maternal age, and maternal education were associated to LBW of the newborn [12–15].In addition, the risk of LBW has been linked to both maternal malnutrition and malaria infection [16]. During the second and third trimesters of pregnancy, intermittent preventative therapy (IPT) for malaria is administered in places where the disease is endemic [17, 18]. Malaria-related birth outcomes can be worsened by inadequate prenatal care attendance, which can also reduce the amount of IPT doses provided [18].

The aforementioned ANC package components have been demonstrated to be cost-effective in reducing the prevalence of LBW elsewhere in SSA [19, 20]. The majority of research on the relationship between ANC and birth weight has been done in high-income countries, even though the prevalence of LBW is higher in low and middle-income countries [21].

The Rwanda Demographic and Health Survey (RDHS) report indicates a prevalence of LBW of 7% [22]. Rwanda’s neonatal mortality rate is estimated to be 18 per 1000 live births, far higher than the United Nations(UN) Sustainable Development Goal 3.2(SDG), which is to reduce neonatal mortality to less than 12 per 1000 live births [23, 24].LBW has been significantly associated with neonatal mortality in resource-limited settings [25, 26]. A study conducted in Rwanda found that 70% of perinatal deaths included low birth weight newborns [27]. For Rwanda to achieve the SDGs in neonatal mortality, determinants of LBW should be assessed to inform the policymakers in the health sector. The 2016 WHO guidelines on ANC recommend at least 8 contacts for every pregnant woman; however, Rwanda still implements the 2001 policy which only recommended 4 visits [22]. According to studies, the quality and content of ANC rather than the number of visits has a stronger influence on maternal and newborn health [28–31]. Quality ANC is when a woman had her first ANC visit within 3months of pregnancy, had 4 or more ANC visits as recommended by WHO [32], and received services components of ANC during the visits(found to be crucial for quality pregnancy care by WHO) [33] by a skilled provider [34]. The choice of this model was adapted from Bollini and colleagues who proposed indicators to help measure quality ANC [35] and referred to a cross-sectional study conducted in India in 2019 [36]. To improve neonatal outcomes in Rwanda, it is imperative to examine the quality and uptake of ANC and their association with LBW.

Few studies have been conducted at the Rwanda country level to examine the associations between quality ANC, health and socioeconomic factors, and LBW [14]. We sought to breach this gap by exploring the associations between quality ANC and potential confounders on LBW.

## Methods

### Study design and data source

This study is a cross-sectional study using secondary data from three waves of the Rwanda Demographic and Health Survey (RDHS). The three waves include RDHS 2010, RDHS 2015, and RDHS 2020. The RDHS is a cross-sectional survey that gathers a sample of households that is nationally representative using a two-stage sampling design. For women in all three waves, response rates were high, topping 99%. The RDHS gathers information on mother and child health during a time frame within the five years prior to the survey. Information on the sample design, sample size, study tools, data collection, getting informed consent, and other related methodologies is presented elsewhere [22]. The datasets of RDHS data were accessible from the Measure DHS website at http://dhsprogram.com/data/available-datasets.cfm.

### Analytic sample

**Fig. 1:**
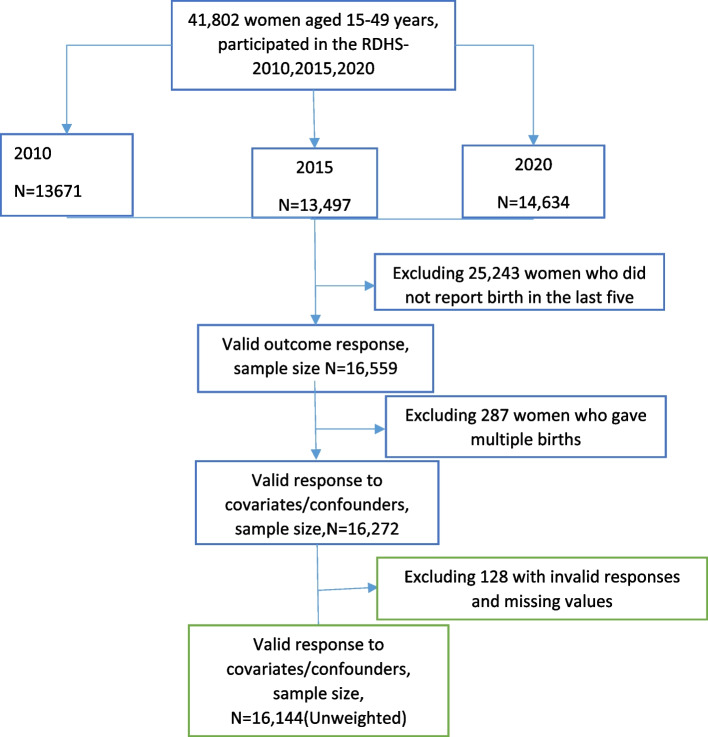
Flow chart of the analytic sample selection

For the purpose of this study, the 2010, 2015, and 2020 RDHS birth recode (BR) datasets were merged based on established guidelines for managing DHS data. Women aged 15 to 49 years old who had a single live birth in the five years prior to each survey and had at least one antenatal care visit answered questions about antenatal care visits(ANC) were included in this sample. Women with missing data or invalid responses to the key exposure, outcome, and possible confounders, such as “don’t know”, were removed. 16,144 of the 41,802 women who took part in the survey met the requirements for inclusion. the flow chart and the analytic sample selection is shown in Fig. 1.

Out of 16,144 births included in this study, information on birth weight was available for 15,560 (96.4%) newborns. Birth weight was obtained through health cards for 4824 (31%) and through maternal recall for 10,736 (69%) newborns.

### Study variables

#### Outcome and exposure

The main outcome was LBW, classified as < 2500 g birth weight. The main exposure variable was quality ANC. Quality ANC is a dichotomous variable, high-quality ANC and low-quality ANC. High-quality ANC is a composite variable which was defined as having had the first ANC visit in the first trimester of pregnancy, had > = 4 ANC visits, having ANC provided by skilled personnel such as a medical doctor, nurse, or midwife and having received all the five interventions during the pregnancy. The five interventions that mark quality ANC are the test of the blood pressure, a urine sample taken, a blood sample taken, giving or bought iron tablets, and receiving a tetanus injection. Low-quality ANC is when the woman missed any quality indicator of ANC.

The definition of the variable “high-quality ANC” was adapted from Bollini P. and colleagues who suggested indicators to assist quantify the quality of antenatal care [35] and referring to a recent study in India [36].

Community level, socioeconomic and demographic factors but also individual level and health service factors were considered as explanatory variables for their relevance in the uptake of ANC and impact on LBW. These factors were adapted from Andersen’s behavioral model, Mosley and Chen [37]. Many studies have made use of Andersen’s behavioral model and the analytical framework by Mosley and Chen to study the determinants of maternal health services utilization and birth outcomes [37–39]. These factors were: Age, type of place of residence (urban, rural), water sources, marital status, preceding birth interval, sex of newborn, maternal education level, household’s wealth index, access to media, birth order, maternal BMI, type of cooking fuel, iron supplementation, receipt of antimalarial treatment. Numerical values like age, birth order and maternal were grouped into categories. Maternal age in years was tabulated into groups (15–19 years, 20–34 years, 35–49 years); the birth order of the baby was into three categories (1st ,2nd -3rd ,4th and above). The preceding birth interval was grouped into two categories (< 24months and > = 24months). utilizing principal component analysis, the household wealth index was created utilizing data on the ownership of durable assets, access to utilities and infrastructure, and dwelling features. 20% of the population of women were divided into five categories depending on their household asset score: poorest, poorer, middle, richer, and richest. Later, three categories (poor, middle class, and rich) were created using these five criteria.

### Statistical analyses

All the statistical analyses were conducted using Stata v17.0 [40]. Descriptive statistics for the sociodemographic characteristics of the study participants were generated **using the** frequency and percentage as shown in Table 1. We used chi-square tests to identify demographic and socio-economic factors associated with the outcome variable. Crude odds ratios were generated using bivariate analyses to determine the odds of each outcome variable with explanatory variables using logistic regression models. Potential factors with *p* < 0.20 were retained for multivariable analysis. When covariates were found to be collinear, using the variance inflation factor**(VIF > 4)**, the variable that was most correlated with the outcome variable of interest was retained. To account for clustering, stratification, and sample weight, we ran all analyses using the survey module “*svyset*” stata commands.

**Table 1:** Baseline characteristics of respondents and the outcome variable

	***N***=16,423	Low birth weight	
Variables	Weighted	<2500g;2.82%	***P***-value
**Quality ANC**			0.2
low	14954(91.10)	710(4.76)	
high	1469(8.94)	56(3.83)	
**Community level factors**	n(%)	n(%)	
**Type of residence**			0.857
urban	2489(15.15)	143(4.59)	
rural	13934(84.85)	623(4.69)	
**Water sources**			0.76
improved	10316(70.70)	466(4.48)	
unimproved	4275(29.30)	191(4.61)	
**Socio-economic&demographic factors**			
** Maternal education**			0.299
no education	2481(15.10)	104(4.29)	
primary	11544(70.29)	564(4.86)	
secondary&higher	2398(14.60)	98(4.2)	
** Married/partnered**			0.008**
no	3007(18.31)	171(5.64)	
yes	13416(81.69)	595(4.46)	
** Access to media**			0.096
not at all	2459(14.98)	133(5.45)	
less than once a week	3856(23.49)	187(4.89)	
at least once a week	10100(61.53)	445(4.4)	
** Wealth index**			<0.001***
poor	7246(44.12)	387(5.33)	
middle	3268(19.90)	150(4.93)	
rich	5909(35.98)	229(3.73)	
** Cooking fuel**			0.162
solid fuel	13706(84.75)	625(4.55)	
non-solid fuel	2467(15.25)	125(5.25)	
**Individual level factors**			
** Maternal age**			0.0025*
15-19	357(2.17)	27(8.29)	
20-34	10976(66.83)	524(4.75)	
35-49	5091(31.00)	215(4.26)	
** Birth order**			<0.001***
1st	3713(22.61)	269(7.32)	
2-3rd	6275(38.21)	263(4.2)	
4th and above	6436(39.19)	234(3.61)	
** Preceding birth interval**			0.026*
< 24months	1947(11.86)	73(3.69)	
>=24months	14476(88.14)	693(4.81)	
** Maternal smoking status**			0.216
no	16279(99.17)	757(4.66)	
yes	136(0.83)	9(7.17)	
** Maternal BMI**			0.365
underweight	6293(38.32)	309(4.88)	
normal	10130(61.68)	457(4.55)	
** Sex of newborn**			<0.001***
male	8370(50.97)	320(3.82)	
female	8053(49.03)	446(5.56)	
**Health service factor**			
** Iron supplementation**			0.576
no	3784(23.04)	174(4.5)	
yes	12639(76.96)	592(4.73)	
** Received anti-malaria treatment**			0.04*
no	10342(86.74)	402(4.06)	
yes	1580(13.26)	73(5.16)	

*p<0.05

**p<0.01

***p<0.001

## Results

Table 1 presents a description of 16,423 mothers of newborns. More than half of the study participants were young women (66.83%) 20–34 years of age. The majority of the study participants resided in rural areas (84.85%), nearly a quarter (70.29%) of the respondents had a primary education level and most (81.69%) were married. In addition, 44.12% of the respondents were in the poor tercile,61.53% of them accessed the media at least once a week and the majority (84.75%) used solid fuel for cooking. Additionally, most of the respondents spaced the birth by 2 years and above (88.14%).

### Quality ANC and LBW

Nearly a tenth (8.94%) of respondents presented high-quality antenatal care, and 2.82% of newborns were born with LBW.

Table 2 shows the details of the interrelationship between various determinants of quality ANC and LBW. Female newborns had 43% increased odds of LBW (aOR:1.43;95% CI:1.18,1.73) compared to male newborns. Rich mothers had 29% decreased odds of LBW(aOR:0.71;95%CI:0.55,0.91) compared to the poor mothers and an increase in the birth order of the newborn reduced the odds of LBW (37% reduced odds for 2nd -3rd birth order aOR:0.63;95%CI:0.49,0.82 and 56% reduced odds for 4th and above birth order aOR:0.44;95%CI:0.32,0.61) compared to the 1st order newborns. High-quality ANC reduced by 33% the odds of LBW(aOR:0.67;95%CI:0.43,1.05) compared to low-quality ANC.

**Table 2:** Crude and adjusted odds ratios for the relationship between quality ANC and LBW

Variables	Low birth weight	Low birth weight
	cOR 95%CI	aOR 95%CI
**Quality ANC**		
low	ref	
high	0.80(0.56,1.13)	0.67(0.43,1.05)
**Community level factors**		
** Type of residence**		
urban	ref	
rural	1.02(0.79,1.33)	--
** Water sources**		
improved	ref	
unimproved	1.03(0.85,1.24)	---
**Socio-economic&demographic factors**		
** Maternal education**		
no education	ref	
primary	1.14(0.90,1.44)	--
secondary&higher	0.98(0.72,1.33)	
** Married/partnered**		
no	ref	
yes	0.78(0.65,0.94)	0.86(0.68,1.09)
** Access to media**		
not at all	ref	
less than once a week	0.89(0.70,1.14)	1.05(0.74,1.48)
at least once a week	0.80(0.64,0.99)	1.02(0.73,1.42)
** Wealth index**		
poor	ref	
middle	0.92(0.75,1.14)	0.82(0.62,1.09)
rich	0.69(0.57,0.83)	0.71(0.55,0.91)***
** Cooking fuel**		
solid fuel	ref	
non-solid fuel	1.16(0.94,1.44)	0.96(0.71,1.29)
**Individual level factors**		
** Maternal age**		
15-19	ref	
20-34	0.55(0.37,0.83)	1.02(0.59,1.76)
35-49	0.49(0.33,0.74)	1.32(0.70,2.47)
** Birth order**		
1st	ref	
2nd-3rd	0.55(0.45,0.68)	0.63(0.49,0.82)***
4th and above	0.47(0.39,0.57)	0.44(0.32,0.61)***
** Preceding birth interval**		
< 24months	ref	
>=24months	1.32(1.03,1.68)	1.04(0.76,1.41)
** Maternal smoking status**		
no	ref	
yes	1.58(0.76,3.30)	---
** Maternal BMI**		
underweight	ref	
normal	0.93(0.79,1.09)	---
** Sex of newborn**		
male	ref	
female	1.48(1.26,1.74)	1.43(1.18,1.73)***
**Health service factor**		
** Iron supplementation**		
no	ref	
yes	1.05(0.87,1.27)	---
** Received anti-malarial treatment**		
no	ref	
yes	1.28(0.99,1.65)	1.29(0.99,1.67)

*p<0.05

** *p*<0.01

***p<0.001 -denotes not considered

**Fig. 2:**
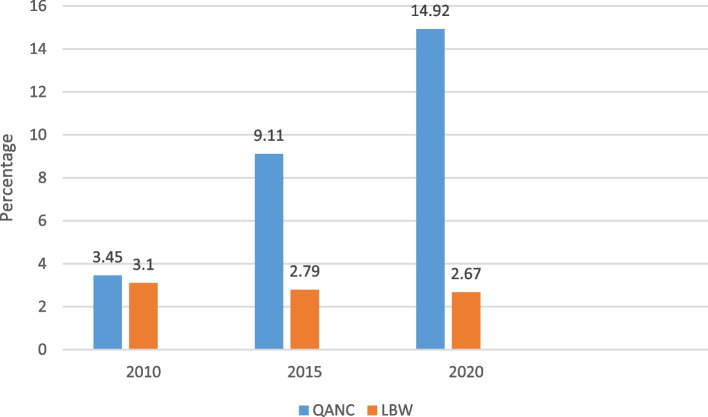
The trend in the percentage of high-quality ANC (QANC) and Low birth weight (LBW)

Figure 2 shows a trend in the percentage of high-quality ANC and LBW. High-quality ANC increased over the last 15 years, and the prevalence of low birth weight decreased.

## Discussion

The current study investigated the association between quality ANC and LBW. We found that 8.94% of the mothers had high-quality ANC visits and 2.82% of the newborns were low birth weight; this result shows that high-quality antenatal care enabled a reduction by 4.18% of the low birth weight to the national average which stands for at 7% according to the report of the most recent survey [22].

Our study showed that high-quality ANC visits was a predictor of LBW. When compared to low-quality ANC visits, high-quality ANC visits had a lower risk of LBW. A recent hospital-based study in Rwanda discovered that women who received four or more ANC visits had a decreased incidence of LBW [41]. Several studies have shown similar conclusions [42–45]. The World Health Organization (WHO) recommends at least four antenatal checkups throughout pregnancy since this is a time when babies are vulnerable to issues such as preterm birth, restricted fetal growth, and congenital infections, all of which increase the likelihood of neonatal death [46]. In addition, attending ANC has been suggested as a possible avenue for mothers and their families to receive information and advice on obstetric care as well as the identification and management of infections such as Malaria, HIV/AIDS, syphilis, and other sexually transmitted diseases that affect the fetus [46]. This emphasizes the necessity of implementing population-based interventions that promote early ANC attendance [44].

Other predictors of LBW include the female gender of newborn. Female neonates were more likely than male neonates to have low birth weight. Findings in Ghana, India, and Brazil corroborate our findings [47–49]. According to Volder and colleagues’ research, paternal birth weight has a considerable impact on boys’ birth weight, but not on girls’ birth weight [50]. LBW was also found to be negatively linked with the rich tercile. Low birth weight neonates were less likely to be delivered by mothers in the rich tercile than by mothers in the poor tercile. Previous research has found that having a higher socioeconomic status lowers the risk of LBW [51–54]. Low birth weight has been linked to poor prenatal nutrition among mothers of lower socioeconomic classes, according to studies [55, 56]. The likelihood of LBW decreases as the newborn’s birth order rises. Several studies have come to the same conclusion [43, 44, 57]. A recent longitudinal study in Germany discovered an increase in birth weight with the newborn’s birth order, implying that the biological intrauterine component is likely to alter mother physiology in favor of later borns and recommending additional research into sibling pregnancies [58].

Our findings demonstrate that a small percentage of women received a high-quality ANC and that their number increased throughout the three waves of surveys. The increase in high-quality ANC played a key role in reducing the prevalence of LBW. However, the prevalence of LBW is still high, future research would examine the effect of several mediator variables such as maternal nutrition during pregnancy on LBW to effectively address this adverse neonatal outcome.

## Strengths and limitations

The study’s use of a nationally representative population-based, combined dataset is a notable strength. We were able to generate a large sample size by merging the three surveys, which allowed us to assess the impact of various factors on LBW with acceptable precision. Because the three DHS used similar sample procedures and questionnaires, used comparable data collection tools, and were planned and implemented by the same institutions, they allowed researchers to look into trends in low birth weight over the past 15 years. This study provided evidence-based information for the decision-makers which can help in the implementation of public health policies regarding ANC improvement and evaluations. Data on key major determinants of maternal healthcare consumption, such as health insurance, was only gathered for the most recent survey, which limited our ability to assess the impact of such variables. Not all potential confounders were included in our study; for instance, gestational age, could have reduced the quality of the results. Variables such as facility readiness, interpersonal relationships between clinicians and women, transportation, and other cultural norms and beliefs that could have influenced a high-quality ANC utilization were not included in this study. Due to the cross-sectional nature of the data, we were only able to investigate relationships rather than causality. Further researchers would conduct a longitudinal study design to assess the causality between ANC and LBW.

## Conclusion

Our findings demonstrate that the use of high-quality ANC has gradually increased. However, the vast majority of the women are still receiving low-quality ANC. The prevalence of LBW has decreased over the years of the surveys, however, it remains high. Addressing the coverage but also the quality of the content in ANC, especially to the poor and primiparous women results in the reduction of the prevalence of LBW. The study revealed that the utilization of high-quality ANC can greatly contribute to lessening LBW and thus neonatal mortality and therefore achieving the SDGs.

## Supplementary Information

**Additional file 1.**

## Data Availability

The data that support the findings of this study are available from the DHS program website http://dhsprogram.com/data/available-datasets.cfm but registration and application is required before access to data is granted.

## Abbreviations

ANC: Antenatal Care
RDHS: Rwanda demographic and Health Survey
LBW: Low Birth Weight
SDGs: Sustainable Development Goals
IPT: Intermittent Preventative Therapy
