# Supplementary material for: Association between quality antenatal care and low birth weight in Rwanda: a cross-sectional study design using the Rwanda demographic and health surveys data
Source: BMC Health Serv Res. 2023 May 30;23:558. doi: 10.1186/s12913-023-09482-9 (PMC10230721; doi:10.1186/s12913-023-09482-9)
Supplement: Supplementary file 1 — Additional file 1. [file 12913_2023_9482_MOESM1_ESM.docx]

**Akaike’s information criterion and Bayesian information criterion**

| Model | N | 11(null) | 11(model) | df | AIC | BIC |
| --- | --- | --- | --- | --- | --- | --- |
|  | 10,868 | -1935.862 | -1899.265 | 15 | 3828.529 | 3937.933 |
